# Association between Epstein-Barr virus serological reactivation and psychological distress: a cross-sectional study of Japanese community-dwelling older adults

**DOI:** 10.18632/aging.204345

**Published:** 2022-10-21

**Authors:** Hirotomo Yamanashi, Shogo Akabame, Jun Miyata, Yukiko Honda, Fumiaki Nonaka, Yuji Shimizu, Seiko Nakamichi, Shin-Ya Kawashiri, Mami Tamai, Kazuhiko Arima, Atsushi Kawakami, Kiyoshi Aoyagi, Takahiro Maeda

**Affiliations:** 1 Department of General Medicine, Nagasaki University Graduate School of Biomedical Sciences, Nagasaki, Japan; 2 Department of Clinical Medicine, Institute of Tropical Medicine, Nagasaki University, Nagasaki, Japan; 3 Department of Island and Community Medicine, Nagasaki University Graduate School of Biomedical Sciences, Nagasaki, Japan; 4 Department of Community Medicine, Nagasaki University Graduate School of Biomedical Sciences, Nagasaki, Japan; 5 Department of Immunology and Rheumatology, Nagasaki University Graduate School of Biomedical Sciences, Nagasaki, Japan; 6 Department of Public Health, Nagasaki University Graduate School of Biomedical Sciences, Nagasaki, Japan

**Keywords:** Epstein-Barr virus, reactivation, early antigen immunoglobulin G, psychological distress, depression

## Abstract

Reactivation of Epstein-Barr virus (EBV) is associated with the etiopathogenesis of a broad spectrum of diseases. This study aimed to investigate the association between psychological distress and EBV serological reactivation among community-dwelling older people and assess the role of sex differences in this association. This population-based cross-sectional survey was conducted among individuals who underwent annual health checkups (N = 2,821; median age 72.4 years). EBV serological reactivation was defined as elevation of EBV early antigen immunoglobulin G titers, and psychological distress was defined as Kessler 6 scores ≥5. Multivariable logistic regression analysis was performed to calculate odds ratios (OR) and 95% confidence intervals (CI) for EBV serological reactivation and psychological distress. EBV serological reactivation and psychological distress were detected in 16.4% and 8.7% of participants, respectively. Women accounted for 71% (328/463) of those with EBV serological reactivation. Multivariable logistic regression analysis showed psychological distress was not significantly associated with EBV serological reactivation among all participants (OR 1.31, 95% CI: 0.95, 1.82; P = 0.102). A sex-stratified multivariable analysis showed a positive association among women (OR 1.45, 95% CI: 1.01, 2.08; P = 0.043), but no association among men. EBV serological reactivation was independently associated with psychological distress in community-dwelling older women. The sex difference in our results warrants further investigation to clarify the physiological mechanisms underlying the association.

## INTRODUCTION

Mental health problems are a major cause of nonfatal diseases and account for an estimated 7.4% of the global burden of disease [1]. In Japan, the lifetime prevalence of mental health problems increased from 17.9% to 22.0% over past decades [2]. The prevalence of psychological distress (e.g., depression and anxiety disorders) in the general population was reported as 5%–27% [3]. Psychological distress has been associated with an increased risk for death [3]. A growing body of research has identified various demographic and socioeconomic factors associated with psychological distress, such as being female [4], unemployed [5], or without a partner [6]. A previous study showed that psychological distress had a U-shaped relationship with age; the prevalence of psychological distress declined from young adulthood to middle age, and then increased in old age [7]. Socioeconomic factors (e.g., low education and widowhood) may contribute to this upturn of psychological distress in older age [7].

Epstein-Barr virus (EBV) is a gamma-herpes virus that is widespread in the adult population and is a known risk factor for the development of various conditions [8–10] such as infectious mononucleosis [11], rheumatoid arthritis [12], systemic erythematosus [13], multiple sclerosis [14], chronic active EBV infection [15], nasopharyngeal carcinoma [16], Burkitt lymphoma [17], and T-cell/NK-cell lymphomas [18]. In contrast to research on autoimmune diseases or carcinogenesis due to EBV, few studies have investigated associations between EBV infection and psychological distress [19, 20]. A cross-sectional study involving 538 adolescents in the United States found increased depressive symptoms was associated with EBV DNA detected in saliva among adolescent women, but not in adolescent men [19]. The sex difference in this association was speculated to relate to sex differences in the immune response to physiological stress during adolescence. A prospective observational study involving pregnant women in late pregnancy found that EBV reactivation, defined as elevated levels of EBV immunoglobulin G (IgG) antibodies, was associated with maternal psychological distress [20]. However, although these studies involving adolescents and pregnant women found an association between EBV infection and psychological distress, no studies have investigated this association in older adults. Furthermore, the link between psychological distress and EBV reactivation and the role of sex differences in this association merit consideration.

The aim of this study was to investigate the association between psychological distress and EBV serological reactivation among older adults and assess the role of sex differences in this association. We hypothesized that there was a link between EBV serological reactivation and psychological distress among older people. Elucidating possible mechanisms underlying psychological distress among older people will benefit public health in the context of global population aging.

## RESULTS

The characteristics of study participants by EBV serological reactivation as defined by early antigen (EA)-IgG titers are shown in Table 1. In total, there were 2,821 participants in this study. The median age was 72.4 years, 1,772 (63%) participants were women, 245 (8.7%) had psychological distress, and EBV serological reactivation was detected in 463 (16.4%) participants. Women had the largest proportion of EBV serological reactivation (71%, 328/463). Compared with the negative group, participants in the positive group were older, more were female, more had lower body mass index (BMI), more had rheumatoid arthritis, more were bereaved or divorced, and more lived alone. A bivariate correlation analysis showed psychological distress was correlated with sex, BMI, marital status (bereaved or divorced), and EBV serological reactivation. EBV serological reactivation was correlated with age, sex, BMI, marital status, and living alone (Table 2). Supplementary Figure 1 shows the scatterplot of EBV EA-IgG titers and age.

**Table 1:** Characteristics of study participants by Epstein-Barr virus serological reactivation (N = 2,821).

**Characteristics**	**Epstein-Barr virus serological reactivation**		
	**Negative**	**Positive**	**P**
No. of participants at risk	2358	463	
Age (years)	73.0 ± 7.3	74.0 ± 7.6	0.008
Sex, female	1444 (61.2)	328 (70.8)	<0.001
Body mass index	23.1 ± 3.3	22.7 ± 3.5	0.012
Dyslipidemia	1061 (45.0)	210 (45.4)	0.887
Modified Charlson Comorbidity Index	0.2 ± 0.5	0.2 ± 0.5	0.563
Stroke	52 (2.2)	17 (3.7)	0.062
Ischemic heart disease	80 (3.4)	15 (3.2)	0.868
Diabetes mellitus	241 (10.2)	44 (9.5)	0.640
Rheumatoid arthritis	18 (0.8)	12 (2.6)	<0.001
Liver disease	125 (5.3)	20 (4.3)	0.382
History of hospitalization in the past one year	268 (11.4)	62 (13.4)	0.215
Marital status			0.012
Married/unmarried/others	1771 (75.1)	322 (69.6)	
Bereaved/divorced	587 (24.9)	141 (30.5)	
Living alone	544 (23.1)	127 (27.4)	0.044
Psychological distress	192 (8.1)	53 (11.5)	0.021

Data presented as mean ± standard deviation or n (%).

**Table 2:** Correlations between psychological distress, Epstein-Barr virus serological reactivation, and other variables.

	**Psychological distress**		**Epstein-Barr virus serological reactivation**	
	**r**	**P**	**r**	**P**
Age (years)	0.03	0.095	0.05	0.008
Sex, female	−0.08	<0.001	−0.07	<0.001
Body mass index	−0.06	0.001	−0.05	0.012
Dyslipidemia	−0.01	0.556	0.00	0.887
Modified Charlson Comorbidity Index	0.03	0.141	0.01	0.563
History of hospitalization in the past one year	0.01	0.487	0.02	0.215
Marital status	0.05	0.010	0.05	0.012
Living alone	0.03	0.092	0.04	0.044
Epstein-Barr virus serological reactivation	0.04	0.021		

In our logistic regression analysis, EBV serological reactivation was significantly associated with psychological distress in all participants (odds ratio [OR] 1.46, 95% confidence interval [CI]: 1.06, 2.01; P = 0.022) (Table 3). An analysis stratified by sex showed that EBV serological reactivation was significantly associated with psychological distress in women (OR 1.53, 95% CI: 1.07, 2.19; P = 0.019) but not in men (OR 0.89, 95% CI: 0.40, 2.00; P = 0.775). Next, we performed a multivariable logistic regression analysis of the association adjusted for age, sex, BMI, dyslipidemia, modified Charlson Comorbidity Index, history of hospitalization in the past 1 year, marital status, and living alone. The association remained significant for women (OR 1.45, 95% CI: 1.01, 2.08; P = 0.043). However, no associations were observed for all participants or in men (OR 1.31, 95% CI: 0.95, 1.82; P = 0.102, OR 0.84, 95% CI: 0.37, 1.90; P = 0.675, respectively).

**Table 3:** Odds ratios (ORs) and 95% confidence intervals (CIs) for Epstein-Barr virus serological reactivation and psychological distress.

	**Epstein-Barr virus serological reactivation**		**P**
	**Negative**	**Positive**	
Psychological distress			
All participants			
No. of participants	2358	463	
No. of cases (%)	192 (8.1)	53 (11.5)	
Crude ORs	1.00	1.46 (1.06, 2.01)	0.022
Multivariable adjusted ORs	1.00	1.31 (0.95, 1.82)	0.102
Women			
No. of participants	1444	328	
No. of cases (%)	139 (9.6)	46 (14.0)	
Crude ORs	1.00	1.53 (1.07, 2.19)	0.019
Multivariable adjusted ORs	1.00	1.45 (1.01, 2.08)	0.043
Men			
No. of participants	914	135	
No. of cases (%)	53 (5.8)	7 (5.2)	
Crude ORs	1.00	0.89 (0.40, 2.00)	0.775
Multivariable adjusted ORs	1.00	0.84 (0.37, 1.90)	0.675

Multivariable model: Adjusted for age, sex, BMI, dyslipidemia, modified Charlson Comorbidity Index, history of hospitalization in the past 1 year, marital status, and living alone.

We stratified participants by the median age (72.4 years) to examine the association by different age groups. The association among the older group (≥72.4 years) was significant for women (OR 1.73, 95% CI: 1.08, 2.75; P = 0.021) (Supplementary Table 1). However, no associations were observed among the other groups.

## DISCUSSION

An important finding in this study was a significant positive association between psychological distress and EBV serological reactivation in community-dwelling older women. A possible explanation for this result was that psychological distress caused stress-induced immunosuppression, which allowed EBV serological reactivation. We also found that the association between EBV serological reactivation and psychological distress in older adults differed by sex.

### Psychological distress, stress-induced immunosuppression, and EBV serological reactivation

Reactivation of EBV has been shown to occur as a result of impaired cellular immune responses evoked by psychosocial stress in old age. Older people may experience a variety of psychosocial stressors, including marital problems [21], social exclusion because of living alone [22], and interpersonal stress related to refusal or being abandoned [23]. A previous study showed that patients with breast cancer who experienced adversity in childhood, such as the death of a parent or serious problems between their parents, had higher antibody titers for EBV than those who did not experience such events [24]. Evidence suggests that social stress for young people (e.g., student examinations) also alters their cellular immunity [25, 26]. A case-control study showed that EBV reactivation, as defined by EBV DNA in saliva, among astronauts who participated in the Space Shuttle mission was increased during the mission compared with the control group [27]. Therefore, a possible explanation for our results is that predisposition to psychological distress may impair the host’s cellular immune response, which leads to EBV serological reactivation [28].

A prospective observational study reported that depressive symptoms in late-gestation mothers were associated with prenatal EBV serological reactivation, as defined by elevation of EA-IgG levels (adjusted OR 2.74, 95% CI: 1.23, 6.08) [20]. In addition, a case-reference study involving pregnant women in the United States with/without pre-pregnancy depression found that depressed women had a higher frequency of EBV serological reactivation (as defined by the EA-IgG antibody) compared with reference participants (48% vs. 30%, P = 0.01) [29]. These results suggested that psychological distress may increase the risk for EBV serological reactivation during pregnancy. Our results were consistent with the results of these previous studies.

### Sex difference in the association between EBV serological reactivation and psychological distress

We found that women accounted for the majority (71%, 328/463) of the EBV serological reactivation group. After adjusting for sex, EBV serological reactivation was only significant in the group of older women. Consistent with our study, previous studies only found an association between EBV reactivation and psychological distress in women [19, 30]. However, an explanation for this sex difference remains to be established. Sex differences in inflammatory, immunological, or endocrinal (e.g., sex hormones) physiology may explain this discrepancy. This finding highlighted the importance of considering sex disparities in further investigations to elucidate the mechanisms underlying the association. As our sample included fewer men than women, we cannot rule out potential random error because of lack of power.

Another possible explanation for our results is a sex difference in cellular immunity modulated by insulin-like growth factor I (IGF-1). Serum IGF-I levels decrease with age [31]. However, in a cohort of healthy adults in the United States, the decline in IGF-I levels was more pronounced in females than in males [31]. Individual susceptibility to mental diseases after experiencing psychosocial stress may also differ between men and women. Mouse experiments showed that the stress response to traumatic brain injury increased the transfer of serum IGF-1 to the brain [32]. In addition, serum IGF-1 was negatively correlated with anxiety behavior in mice measured 1 week after stress from the elevated plus maze [32]. In a cohort of 2,686 Dutch adults, IGF-1 was associated with stressful life event experiences [32]. An observational cohort study involving 8,791 Japanese pregnant women found that women with high serum IGF-1 levels in early pregnancy were less likely to develop postpartum depression than women with low serum IGF-1 levels [33]. These results implied IGF-1 is involved in mood homeostasis and has a protective role in psychological distress. IGF-1 regulates the immune function in various aspects of T cells, B cells, and monocytes through binding to the IGF-1 receptor and may therefore act protectively in EBV serological reactivation. In older age, women have a more pronounced decline in IGF-1 levels than men. Therefore, the stress-induced immune response modified by IGF-1 may explain the sex difference in the association between psychological distress and EBV serological reactivation.

We also found a strong association in older women after age stratification, which supported the EBV serological reactivation mechanism being due to psychological distress. Older participants tended to have lower IGF-1 levels and were more sensitive to stress, which may have increased their risk for altered immunity modulation. However, no such reaction was observed in men, and the association was strong in women, which supported the existence of a clear sex difference.

### Study strengths and limitations

A major strength of this study was the large number of participants (N = 2,821). Another strength was our use of EBV EA-IgG instead of EBV viral capsid antigen (VCA) IgG. The seropositivity of EBV VCA IgG for children aged 5–9 years exceeds 90% in east Asia, and is especially high in Japanese children (100% in 1968) [34]. A recent study also showed that the VCA IgG was almost 100% positive among patients with inflammatory bowel disease aged ≥60 years [35]. In terms of the seropositivity difference among generations, our study cohort comprised a generation born between 1924 and 1959. Compared with Western populations, Asian children tend to be infected early in life (i.e., before 1 year of age) and >90% of children aged 5–9 years are infected [36]. In addition, the recent time trend of EBV VCA IgG seropositivity among children in Japan showed a gradual decrease, but epidemiology data for Japan suggested this was expected to be ≥90% between 1924 and 1959 [36]. Because EBV VCA IgG is ubiquitous among Japanese older people, the detection of EBV serological reactivation using EBV VCA IgG titers may have had a ceiling effect, meaning that most participants among the older population remained positive. Therefore EBV EA-IgG may be a worthwhile marker to include in further studies of EBV in older adults.

Some limitations of this study need to be considered. First, our cross-sectional design meant we were unable to establish cause–effect relationships. Second, there is potential for random error because of the sample size, which might have led to the sex difference in the association between EBV serological reactivation and psychological distress. Third, there might have been some diagnostic uncertainty. We defined psychological distress using an interview based on the K6, not a self-administered questionnaire. Fourth, we had no information about diagnosis or treatment of mental diseases. These factors may be potential residual confounders. Fifth, although we defined EBV serological reactivation using EBV EA-IgG, we could not assess EBV DNA to detect reactivation. Sixth, we cannot completely rule out the existence of primary EBV infection in our study. However, because of our large sample size and the large proportion of older adults infected in early life in Japan [36], the presence of potential cases of primary EBV infection is unlikely to change our conclusions. Finally, we cannot rule out psychological distress due to chronic somatic diseases, especially autoimmune diseases such as systemic lupus erythematosus and antiphospholipid syndrome. However, our results were adjusted for chronic illnesses, including rheumatoid arthritis.

## CONCLUSIONS

EBV serological reactivation was independently associated with psychological distress, with this association only observed among women. The prominent effect of psychological problems in older people needs further investigation to clarify their role in EBV serological reactivation and elucidate the sex difference in the association.

## MATERIALS AND METHODS

### Study setting and participants

We conducted a cross-sectional survey in Goto City, which is located in the Goto Islands, which comprise the western archipelago of Nagasaki Prefecture. The population of Goto City was 37,327 people in 2015, of which 36.8% were aged 65 years and over. In Japan, screening and treatment for non-communicable diseases is conducted based on the Health and Welfare for the Aged Act, and Goto City conducts health examinations for adults aged 40 years and over who live in the community. These health examinations are held at community centers in all districts of Goto City from April to September every year.

The Nagasaki Islands study was conducted with the cooperation of Goto City, and targeted chronic diseases such as atherosclerosis, cerebrovascular diseases, rheumatoid arthritis, frailty, and sarcopenia. We distributed flyers to all family units in the study areas, which explained our study as an additional medical checkup free of charge to the population aged 60 years or older. The participant recruitment period was from 2017 to 2019. We approached all adults who underwent a medical examination. We excluded 578 individuals because they were younger than 60 years (n = 516) or had missing data for EBV EA-IgG (n = 58), K6 score (n = 2), medication history of diabetes mellitus (n = 1), or low-density lipoprotein cholesterol (n = 1). This left 2,821 participants (1,049 men and 1,772 women) aged 60–94 years for inclusion in this study.

### Examinations

The researchers and trained research assistants administered an interview to obtain information on: participants’ past medical history of stroke (yes or no), ischemic heart disease (yes or no), diabetes mellitus (yes or no), dyslipidemia (yes or no), joint pain (yes or no), history of hospitalization in the past 1 year (yes or no), marital status (married/unmarried/other or bereaved/divorced), and number of household members.

Body weight and height were measured with participants wearing light-weight clothing and without shoes, and the BMI of each participant was calculated. Blood samples were collected using heparin sodium and a siliconized tube at the time of clinical examination. Most participants may have fasted because they were instructed to fast before undergoing their health checkup. Serum concentrations of low-density lipoprotein cholesterol, high-density lipoprotein cholesterol, triglycerides, aspartate aminotransferase, alanine aminotransferase, anti-cyclic citrullinated peptide (anti-CCP) antibodies, and HbA1c were measured by standard laboratory procedures.

Several variables were defined or calculated based on the questionnaire and laboratory results. Diabetes mellitus was defined as an HbA1c concentration ≥6.5% or current use of hypoglycemic drugs. Rheumatoid arthritis was defined as an anti-CCP antibodies concentration ≥4.5 U/mL and presence of joint pain. Liver disease was defined as an aspartate aminotransferase or alanine aminotransferase concentration ≥40 U/L. Dyslipidemia was defined as a low-density lipoprotein cholesterol concentration ≥140 mg/dL, high-density lipoprotein cholesterol concentration <40 mg/dL, triglycerides concentration ≥150 mg/dL, or use of lipid-lowering drugs. As an indicator of chronic illnesses, we used the modified Charlson Comorbidity Index to obtain the sum of the number of chronic illnesses (stroke, ischemic heart disease, diabetes mellitus, rheumatoid arthritis, and liver disease) [37].

### Measurement of EBV EA-IgG

We defined EBV EA-IgG titers as an indicator of EBV serological reactivation with the below-mentioned rationale. EBV enters memory B cells and escapes the immune response by restricting the expression of the virus’s own genes [9]. EBV maintains latent infection on infected B cells and reactivates during the lysis cycle. The EBV EA-IgG response is considered indirect evidence of the initiation of lytic replication [38]. In this process, EBV infection elicits an IgG response to VCA, followed by an IgG response to early antigen. EBV EA-IgG increases during the first 3–4 weeks and is detected from a few months to up to 2 years after infection, but high titers have also been seen during reactivation [13, 39]. In contrast to EBV EA-IgG, VCA IgG remains generally detectable for life after acute EBV infection, and antibodies against EBV nuclear antigen remain expressed during the latent phase of the infection [40]. Therefore, in the present study, the seropositivity of EBV EA-IgG was defined as an indicator of serological reactivation of the lytic cycle of EBV infection.

An enzyme immunoassay kit (Denka Inc., Niigata, Japan) was used for EBV EA-IgG detection. This kit detects anti-EA antibodies that reflect a diffuse pattern: EA (D) IgG. Standard calibrators were used in each assay to calculate index values/optical density ratios, which served as a semiquantitative measure of antibody levels. All assays met predetermined quality control measures based on positive, negative, and blank controls. The results were based on index cutoff values: ≤0.5 was considered negative, 0.5–0.9 was considered equivocal, and ≥1.0 was considered positive according to the manufacturer’s instructions. We defined EBV serological reactivation using the EBV EA-IgG index cutoff value of 1.0.

### Kessler 6 (K6)

The K6 comprises six items based on the item response theory and is used for screening psychological distress [41, 42]. The test has good reliability and validity, and the validity of the Japanese version has been established [43, 44]. Participants respond to the question: “During the past 30 days, about how often did you feel: 1) nervous, 2) hopeless, 3) restless or fidgety, 4) so depressed that nothing could cheer you up, 5) that everything was an effort, and 6) worthless?” Responses are on a 5-point scale (0 = none of the time to 4 = all of the time). We calculated the sum of the reported scores (range: 0–24) to obtain participants’ K6 scores. A higher score indicates greater severity. Based on a previous study that screened psychological distress in a Japanese population [42], we defined a K6 score of ≤4 as no psychological distress, and a score ≥5 as psychological distress.

### Statistical analyses

Differences in mean values or proportions of variables by EBV serological reactivation were analyzed using Student’s t-tests for continuous variables (age, BMI, modified Charlson Comorbidity Index), and McNemar chi-square tests for categorical variables (sex, dyslipidemia, stroke, ischemic heart disease, diabetes mellitus, rheumatoid arthritis, liver disease, history of hospitalization in the past 1 year, marital status, living alone, and psychological distress). There were no missing values. We performed a Pearson’s bivariate correlation analysis to examine associations between EBV serological reactivation and other variables, and psychological distress and other variables. A scatterplot showing EBV EA-IgG and age for all participants and by sex was prepared.

Next, we performed logistic regression analysis and calculated ORs and 95% CIs for EBV serological reactivation and psychological distress. In the multivariable logistic regression analysis, adjustments were made *a priori* for variables based on previous studies [7, 45–49]; age (continuous variable), sex (dichotomous variable), BMI (continuous variable), dyslipidemia (dichotomous variable: no, yes), modified Charlson Comorbidity Index (continuous variable), history of hospitalization in the past 1 year (dichotomous variable: no, yes), marital status (dichotomous variable: married/unmarried/other or bereaved/divorced; 0, 1, respectively), and living alone (dichotomous variable: no, yes). We conducted logistic regression analysis stratified by sex to examine any sex-based differences. Because the correlation coefficient between EBV serological reactivation and age was positive, we considered it was worth investigating associations linked to age. Therefore, we stratified participants into younger and older groups by the median age (72.4 years). We used logistic regression analysis to examine EBV serological reactivation and psychological distress stratified by age. All analyses were two-tailed, and P-values < 0.05 were regarded as statistically significant. Statistical analyses were performed using STATA® version 14.0 (StataCorp, College Station, TX, USA).

## Supplementary Material

Supplementary Figure 1

Supplementary Table 1

## References

[1] Becker AE, Kleinman A. Mental health and the global agenda. N Engl J Med. 2013; 369:66–73. 10.1056/NEJMra1110827 23822778

[2] Ishikawa H, Tachimori H, Takeshima T, Umeda M, Miyamoto K, Shimoda H, Baba T, Kawakami N. Prevalence, treatment, and the correlates of common mental disorders in the mid 2010’s in Japan: The results of the world mental health Japan 2nd survey. J Affect Disord. 2018; 241:554–62. 10.1016/j.jad.2018.08.050 30153639

[3] Davison KM, Lung Y, Lin SL, Tong H, Kobayashi KM, Fuller-Thomson E. Psychological distress in older adults linked to immigrant status, dietary intake, and physical health conditions in the Canadian Longitudinal Study on Aging (CLSA). J Affect Disord. 2020; 265:526–37. 10.1016/j.jad.2020.01.024 32090781

[4] Matud MP, Bethencourt JM, Ibáñez I. Gender differences in psychological distress in Spain. Int J Soc Psychiatry. 2015; 61:560–8. 10.1177/0020764014564801 25534418

[5] Thomas C, Benzeval M, Stansfeld S. Psychological distress after employment transitions: the role of subjective financial position as a mediator. J Epidemiol Community Health. 2007; 61:48–52. 10.1136/jech.2005.044206 17183015 PMC2465589

[6] Hope S, Rodgers B, Power C. Marital status transitions and psychological distress: longitudinal evidence from a national population sample. Psychol Med. 1999; 29:381–9. 10.1017/s0033291798008149 10218928

[7] Schieman S, Van Gundy K, Taylor J. Status, role, and resource explanations for age patterns in psychological distress. J Health Soc Behav. 2001; 42:80–96. 11357720

[8] Houen G, Trier NH. Epstein-Barr Virus and Systemic Autoimmune Diseases. Front Immunol. 2021; 11:587380. 10.3389/fimmu.2020.587380 33488588 PMC7817975

[9] Macsween KF, Crawford DH. Epstein-Barr virus-recent advances. Lancet Infect Dis. 2003; 3:131–40. 10.1016/s1473-3099(03)00543-7 12614729

[10] Fugl A, Andersen CL. Epstein-Barr virus and its association with disease - a review of relevance to general practice. BMC Fam Pract. 2019; 20:62. 10.1186/s12875-019-0954-3 31088382 PMC6518816

[11] Lennon P, Crotty M, Fenton JE. Infectious mononucleosis. BMJ. 2015; 350:h1825. 10.1136/bmj.h1825 25899165

[12] Balandraud N, Roudier J. Epstein-Barr virus and rheumatoid arthritis. Joint Bone Spine. 2018; 85:165–70. 10.1016/j.jbspin.2017.04.011 28499895

[13] Jog NR, Young KA, Munroe ME, Harmon MT, Guthridge JM, Kelly JA, Kamen DL, Gilkeson GS, Weisman MH, Karp DR, Gaffney PM, Harley JB, Wallace DJ, et al. Association of Epstein-Barr virus serological reactivation with transitioning to systemic lupus erythematosus in at-risk individuals. Ann Rheum Dis. 2019; 78:1235–41. 10.1136/annrheumdis-2019-215361 31217170 PMC6692217

[14] Bjornevik K, Cortese M, Healy BC, Kuhle J, Mina MJ, Leng Y, Elledge SJ, Niebuhr DW, Scher AI, Munger KL, Ascherio A. Longitudinal analysis reveals high prevalence of Epstein-Barr virus associated with multiple sclerosis. Science. 2022; 375:296–301. 10.1126/science.abj8222 35025605

[15] Fujiwara S, Nakamura H. Chronic Active Epstein-Barr Virus Infection: Is It Immunodeficiency, Malignancy, or Both? Cancers (Basel). 2020; 12:3202. 10.3390/cancers12113202 33143184 PMC7692233

[16] Chen WJ, Xu WN, Wang HY, Chen XX, Li XQ, Xie SH, Lin DF, Cao SM. Plasma Epstein-Barr virus DNA and risk of nasopharyngeal carcinoma in a prospective seropositive population. BMC Cancer. 2021; 21:651. 10.1186/s12885-021-08408-0 34074258 PMC8168313

[17] Pannone G, Zamparese R, Pace M, Pedicillo MC, Cagiano S, Somma P, Errico ME, Donofrio V, Franco R, De Chiara A, Aquino G, Bucci P, Bucci E, et al. The role of EBV in the pathogenesis of Burkitt’s Lymphoma: an Italian hospital based survey. Infect Agent Cancer. 2014; 9:34. 10.1186/1750-9378-9-34 25364378 PMC4216353

[18] Saleem A, Natkunam Y. Extranodal NK/T-Cell Lymphomas: The Role of Natural Killer Cells and EBV in Lymphomagenesis. Int J Mol Sci. 2020; 21:1501. 10.3390/ijms21041501 32098335 PMC7073055

[19] Ford JL, Stowe RP. Depressive symptoms are associated with salivary shedding of Epstein-Barr virus in female adolescents: The role of sex differences. Psychoneuroendocrinology. 2017; 86:128–33. 10.1016/j.psyneuen.2017.09.009 28954244 PMC5905709

[20] Zhu P, Chen YJ, Hao JH, Ge JF, Huang K, Tao RX, Jiang XM, Tao FB. Maternal depressive symptoms related to Epstein-Barr virus reactivation in late pregnancy. Sci Rep. 2013; 3:3096. 10.1038/srep03096 24172862 PMC3813936

[21] Jaremka LM, Glaser R, Malarkey WB, Kiecolt-Glaser JK. Marital distress prospectively predicts poorer cellular immune function. Psychoneuroendocrinology. 2013; 38:2713–9. 10.1016/j.psyneuen.2013.06.031 23880114 PMC3812415

[22] Glaser R, Kiecolt-Glaser JK, Speicher CE, Holliday JE. Stress, loneliness, and changes in herpesvirus latency. J Behav Med. 1985; 8:249–60. 10.1007/BF00870312 3003360

[23] Fagundes CP, Jaremka LM, Glaser R, Alfano CM, Povoski SP, Lipari AM, Agnese DM, Yee LD, Carson WE 3rd, Farrar WB, Malarkey WB, Chen M, Kiecolt-Glaser JK. Attachment anxiety is related to Epstein-Barr virus latency. Brain Behav Immun. 2014; 41:232–8. 10.1016/j.bbi.2014.04.002 24945717 PMC4304069

[24] Fagundes CP, Glaser R, Malarkey WB, Kiecolt-Glaser JK. Childhood adversity and herpesvirus latency in breast cancer survivors. Health Psychol. 2013; 32:337–44. 10.1037/a0028595 22746260 PMC4008097

[25] Glaser R, Pearl DK, Kiecolt-Glaser JK, Malarkey WB. Plasma cortisol levels and reactivation of latent Epstein-Barr virus in response to examination stress. Psychoneuroendocrinology. 1994; 19:765–72. 10.1016/0306-4530(94)90023-x 7991763

[26] Glaser R, Friedman SB, Smyth J, Ader R, Bijur P, Brunell P, Cohen N, Krilov LR, Lifrak ST, Stone A, Toffler P. The differential impact of training stress and final examination stress on herpesvirus latency at the United States Military Academy at West Point. Brain Behav Immun. 1999; 13:240–51. 10.1006/brbi.1999.0566 10469525

[27] Pierson DL, Stowe RP, Phillips TM, Lugg DJ, Mehta SK. Epstein-Barr virus shedding by astronauts during space flight. Brain Behav Immun. 2005; 19:235–42. 10.1016/j.bbi.2004.08.001 15797312

[28] McClure HH, Martinez CR, Snodgrass JJ, Eddy JM, Jiménez RA, Isiordia LE, McDade TW. Discrimination-related stress, blood pressure and Epstein-Barr virus antibodies among Latin American immigrants in Oregon, US. J Biosoc Sci. 2010; 42:433–61. 10.1017/S0021932010000039 20178683

[29] Haeri S, Johnson N, Baker AM, Stuebe AM, Raines C, Barrow DA, Boggess KA. Maternal depression and Epstein-Barr virus reactivation in early pregnancy. Obstet Gynecol. 2011; 117:862–6. 10.1097/AOG.0b013e31820f3a30 21422857

[30] McDade TW, Stallings JF, Angold A, Costello EJ, Burleson M, Cacioppo JT, Glaser R, Worthman CM. Epstein-Barr virus antibodies in whole blood spots: a minimally invasive method for assessing an aspect of cell-mediated immunity. Psychosom Med. 2000; 62:560–8. 10.1097/00006842-200007000-00015 10949102

[31] O’Connor KG, Tobin JD, Harman SM, Plato CC, Roy TA, Sherman SS, Blackman MR. Serum levels of insulin-like growth factor-I are related to age and not to body composition in healthy women and men. J Gerontol A Biol Sci Med Sci. 1998; 53:M176–82. 10.1093/gerona/53a.3.m176 9597048

[32] Santi A, Bot M, Aleman A, Penninx BW, Aleman IT. Circulating insulin-like growth factor I modulates mood and is a biomarker of vulnerability to stress: from mouse to man. Transl Psychiatry. 2018; 8:142. 10.1038/s41398-018-0196-5 30068974 PMC6070549

[33] Adachi S, Tokuda N, Kobayashi Y, Tanaka H, Sawai H, Shibahara H, Takeshima Y, Shima M, and Japan Environment and Children’s Study Group. Association between the serum insulin-like growth factor-1 concentration in the first trimester of pregnancy and postpartum depression. Psychiatry Clin Neurosci. 2021; 75:159–65. 10.1111/pcn.13200 33459438 PMC8248044

[34] Hinuma Y, Ota-Hatano R, Suto T, Numazaki Y. High incidence of Japanese infants with antibody to a herpes-type virus associated with cultured Burkitt lymphoma cells. Jpn J Microbiol. 1969; 13:309–11. 10.1111/j.1348-0421.1969.tb00472.x 4899489

[35] Miura M, Shimizu H, Saito D, Miyoshi J, Matsuura M, Kudo T, Hirayama D, Yoshida M, Arai K, Iwama I, Nakase H, Shimizu T, Hisamatsu T. Multicenter, cross-sectional, observational study on Epstein-Barr viral infection status and thiopurine use by age group in patients with inflammatory bowel disease in Japan (EBISU study). J Gastroenterol. 2021; 56:1080–91. 10.1007/s00535-021-01832-w 34591171

[36] Takeuchi K, Tanaka-Taya K, Kazuyama Y, Ito YM, Hashimoto S, Fukayama M, Mori S. Prevalence of Epstein-Barr virus in Japan: trends and future prediction. Pathol Int. 2006; 56:112–6. 10.1111/j.1440-1827.2006.01936.x 16497243

[37] Charlson ME, Pompei P, Ales KL, MacKenzie CR. A new method of classifying prognostic comorbidity in longitudinal studies: development and validation. J Chronic Dis. 1987; 40:373–83. 10.1016/0021-9681(87)90171-8 3558716

[38] Hess RD. Routine Epstein-Barr virus diagnostics from the laboratory perspective: still challenging after 35 years. J Clin Microbiol. 2004; 42:3381–7. 10.1128/JCM.42.8.3381-3387.2004 15297472 PMC497621

[39] De Paschale M, Clerici P. Serological diagnosis of Epstein-Barr virus infection: Problems and solutions. World J Virol. 2012; 1:31–43. 10.5501/wjv.v1.i1.31 24175209 PMC3782265

[40] Sanosyan A, Daien C, Nutz A, Bollore K, Bedin AS, Morel J, Zimmermann V, Nocturne G, Peries M, Guigue N, Gottenberg JE, Van de Perre P, Mariette X, Tuaillon E. Discrepancy of Serological and Molecular Patterns of Circulating Epstein-Barr Virus Reactivation in Primary Sjögren’s Syndrome. Front Immunol. 2019; 10:1153. 10.3389/fimmu.2019.01153 31191532 PMC6549440

[41] Kessler RC, Andrews G, Colpe LJ, Hiripi E, Mroczek DK, Normand SL, Walters EE, Zaslavsky AM. Short screening scales to monitor population prevalences and trends in non-specific psychological distress. Psychol Med. 2002; 32:959–76. 10.1017/s0033291702006074 12214795

[42] Sakurai K, Nishi A, Kondo K, Yanagida K, Kawakami N. Screening performance of K6/K10 and other screening instruments for mood and anxiety disorders in Japan. Psychiatry Clin Neurosci. 2011; 65:434–41. 10.1111/j.1440-1819.2011.02236.x 21851452

[43] Furukawa TA, Kawakami N, Saitoh M, Ono Y, Nakane Y, Nakamura Y, Tachimori H, Iwata N, Uda H, Nakane H, Watanabe M, Naganuma Y, Hata Y, et al. The performance of the Japanese version of the K6 and K10 in the World Mental Health Survey Japan. Int J Methods Psychiatr Res. 2008; 17:152–8. 10.1002/mpr.257 18763695 PMC6878390

[44] Cornelius BL, Groothoff JW, van der Klink JJ, Brouwer S. The performance of the K10, K6 and GHQ-12 to screen for present state DSM-IV disorders among disability claimants. BMC Public Health. 2013; 13:128. 10.1186/1471-2458-13-128 23402478 PMC3575398

[45] Hegeman JM, van Fenema EM, Comijs HC, Kok RM, van der Mast RC, de Waal MW. Effect of chronic somatic diseases on the course of late-life depression. Int J Geriatr Psychiatry. 2017; 32:779–87. 10.1002/gps.4523 27273023

[46] Triolo F, Harber-Aschan L, Belvederi Murri M, Calderón-Larrañaga A, Vetrano DL, Sjöberg L, Marengoni A, Dekhtyar S. The complex interplay between depression and multimorbidity in late life: risks and pathways. Mech Ageing Dev. 2020; 192:111383. 10.1016/j.mad.2020.111383 33045250

[47] Alexopoulos GS. Mechanisms and treatment of late-life depression. Transl Psychiatry. 2019; 9:188. 10.1038/s41398-019-0514-6 31383842 PMC6683149

[48] Kerr JR. Epstein-Barr virus (EBV) reactivation and therapeutic inhibitors. J Clin Pathol. 2019; 72:651–8. 10.1136/jclinpath-2019-205822 31315893

[49] Palmer S, Vecchio M, Craig JC, Tonelli M, Johnson DW, Nicolucci A, Pellegrini F, Saglimbene V, Logroscino G, Fishbane S, Strippoli GF. Prevalence of depression in chronic kidney disease: systematic review and meta-analysis of observational studies. Kidney Int. 2013; 84:179–91. 10.1038/ki.2013.77 23486521

