# Supplementary figures and images for: Association between Epstein-Barr virus serological reactivation and psychological distress: a cross-sectional study of Japanese community-dwelling older adults
Source: Aging (Albany NY). 2022 Oct 21;14(20):8258–69. doi: 10.18632/aging.204345 (PMC9648801; doi:10.18632/aging.204345)

SUPPLEMENTARY FIGURE

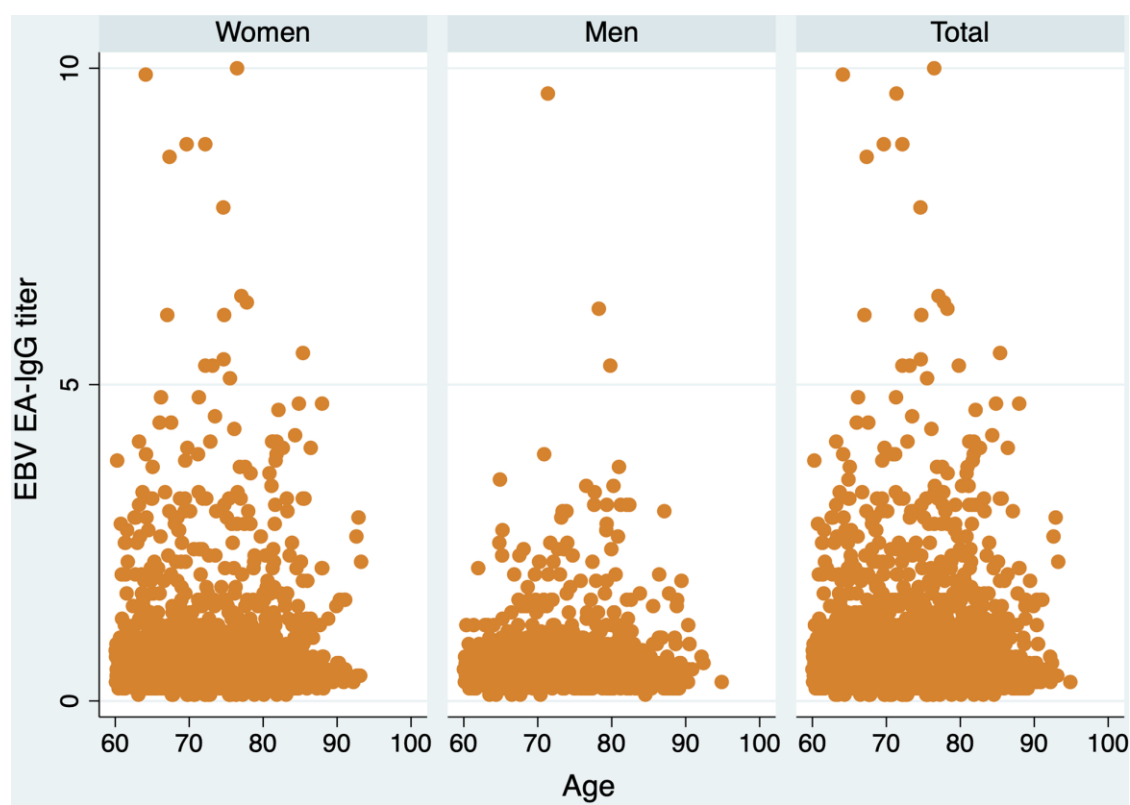

Supplementary Figure 1. Scatterplot of EBV EA-IgG titers and age.

Supplement: Supplementary Figure 1 [file aging-14-204345-s001.pdf]
