# Supplementary material for: Association between Epstein-Barr virus serological reactivation and psychological distress: a cross-sectional study of Japanese community-dwelling older adults
Source: Aging (Albany NY). 2022 Oct 21;14(20):8258–69. doi: 10.18632/aging.204345 (PMC9648801; doi:10.18632/aging.204345)
Supplement: Supplementary Table 1 [file aging-14-204345-s002.pdf]

SUPPLEMENTARY TABLE

Supplementary Table 1. Odds ratios (ORs) and 95% confidence intervals (CIs) for Epstein-Barr virus serological reactivation and psychological distress by age group.

	Epstein-Barr virus serological reactivation			Epstein-Barr virus serological reactivation		
	**Negative**	**Positive**	**P**	**Negative**	**Positive**	**P**
	Younger age group (60-72)			Older age group (72-94)		
Psychological distress						
All participants						
No. of participants	1197	214		1161	249	
No. of cases (%)	87 (7.3)	20 (9.4)		105 (9.0)	33 (13.3)	
Crude ORs	1.00	1.32 (0.79, 2.19)	0.292	1.00	1.54 (1.01, 2.33)	0.044
Multivariable adjusted ORs	1.00	1.18 (0.70, 1.98)	0.530	1.00	1.41 (0.93, 2.16)	0.109
Women						
No. of participants	725	156		719	172	
No. of cases (%)	63 (8.7)	16 (10.3)		76 (10.6)	30 (17.4)	
Crude ORs	1.00	1.20 (0.67, 2.14)	0.535	1.00	1.79 (1.13, 2.83)	0.013
Multivariable adjusted ORs	1.00	1.07 (0.59, 1.94)	0.820	1.00	1.73 (1.08, 2.75)	0.021
Men						
No. of participants	472	58		442	77	
No. of cases (%)	24 (5.1)	4 (6.9)		29 (6.6)	3 (3.9)	
Crude ORs	1.00	1.38 (0.46, 4.14)	0.562	1.00	0.58 (0.17, 1.94)	0.375
Multivariable adjusted ORs	1.00	1.40 (0.46, 4.27)	0.558	1.00	0.55 (0.16, 1.86)	0.334

Multivariable model: Adjusted for age, sex, BMI, dyslipidemia, modified Charlson Comorbidity Index, history of hospitalization in the past 1 year, marital status, and living alone.
